# High glutamate permeability and distal localization of Best1 channel in CA1 hippocampal astrocyte

**DOI:** 10.1186/1756-6606-6-54

**Published:** 2013-12-09

**Authors:** Hyungju Park, Kyung-Seok Han, Soo-Jin Oh, Seonmi Jo, Junsung Woo, Bo-Eun Yoon, C Justin Lee

**Affiliations:** 1 Center for Neural Science and WCI Center for Functional Connectomics, Korea Institute of Science and Technology (KIST), Seoul, Korea; 2 Neuroscience Program, University of Science and Technology (UST), Daejeon, Korea; 3 KU-KIST Graduate School of Converging Science & Technology, Seoul, Korea; 4 Department of Biological Science, Korea Advanced Institute of Science and Technology (KAIST), Daejeon, Korea; 5 Department of Nanobiomedical Science, Dankook University, Cheonan, Korea

**Keywords:** Astrocyte, Bestrophin-1, Glutamate, Anion channel

## Abstract

**Background:**

Glutamate is the major neurotransmitter that mediates a principal form of excitatory synaptic transmission in the brain. From the presynaptic terminals of neurons, glutamate is released upon exocytosis of the glutamate-packaged vesicles. In recent years, astrocytes are also known to release glutamate via various routes to modulate synaptic transmission. In particular, we have characterized a glutamate-permeable Ca^2+^-activated anion channel encoded by *Bestrophin 1* gene (Best1) that is responsible for Ca^2+^-dependent, channel-mediated glutamate release in astrocyte. Best1 channel contains a large pore that is readily permeable to large molecules such as glutamate and GABA. In those studies we obtained permeability ratio of glutamate to Cl^-^ in heterologously expressed mouse Best1 in HEK293T cells and in endogenously expressed mouse Best1 in cultured astrocytes. However, up to now, glutamate permeability of the native Best1 channel *in vivo* has not been reported.

**Findings:**

In whole-cell recordings of CA1 hippocampal astrocytes, we found that opening of Best1 channel upon activation of a Gq-coupled GPCR, protease-activated receptor 1 (PAR1) generated the anion current carried by glutamate via Ca^2+^ increase. This Ca^2+^-evoked glutamate-mediated anion current was unaffected by pretreatment of the inhibitors for a gap junction hemi-channel or Ca^2+^-activated K^+^ conductance. This astrocytic anion conductance carried by glutamate was mediated by Best1 channel expression in CA1 hippocampal astrocytes, because *Best1* knock-down by shRNA expression eliminated astrocytic glutamate conductance by PAR-1 activation. However, we found that these astrocytes showed a deviation in reversal potential of Best1-mediated current from the predicted value. By performing dual patch recording, we concluded that the deviation of reversal potential is due to incomplete space clamping arising from extremely leaky membrane (input resistance ranging 1–3 MΩ), very low length constant of astrocytic processes, and the localization of Best1 channel in distal microdomains near synapses. Based on the relative shift of reversal potentials by ion substitutions, we estimated the permeability ratio of glutamate and Cl^-^ (P_glutamate_/P_Cl_) as 0.53.

**Conclusions:**

Our study shows that Best1, located at the microdomains near the synaptic junctions, has a significantly high permeability to glutamate *in vivo,* serving as the prominent glutamate-releasing channel in astrocytes, mediating the release of various gliotransmitters in the brain, and playing an important role in modulating synaptic transmission.

## Background

Astrocytes modulate neuronal function by releasing glutamate in response to neuronal activity [1,2]. Astrocytically released glutamate has been shown to activate neighboring neuronal glutamate receptors such as N-methyl-D-aspartic acid receptor (NMDAR) which is a critical player for diverse neuronal function [3-5]. Thus, delineation of underlying mechanisms of glutamate release from astrocytes has received much attention.

Recently, it has been reported that astrocytic glutamate is released through glutamate-permeable channels, such as Ca^2+^-activated anion channel, Best1 and TREK-1 containing two pore potassium channel [4,5]. In particular, Best1 is responsible for the Ca^2+^-dependent, channel-mediated glutamate release from astrocytes [4]. Due to the preferential subcellular localization of Best1 at the microdomain near neuronal synaptic sites [4], it is believed that glutamate released through Best1 specifically targets postsynaptically localized neuronal NMDA receptors [3] and plays a critical role in modulating synaptic function [3].

The unique property of Best1 in mediating channel-mediated glutamate release originates from its pore that allows large ions and molecules to pass through [6,7]. Best1 has been shown to be permeable to large ions and molecules with a significant permeability ratio of anion to Cl^-^ (P_x_/P_Cl_): GABA (0.19) [6], isothionate (0.47) [7], bicarbonate (0.44) [8], gluconate (0.4) [9]. Despite the strong evidence for functional expression, physiological importance, and unique biophysical property of Best1, there has been no description or estimation of glutamate permeability of Best1 *in vivo*[4,7]. To address this issue, we performed an electrophysiological measurement of whole-cell anion conductance from astrocytes in acute hippocampal slice to identify a Ca^2+^-dependent glutamate conductance mediated by Best1.

## Results and discussions

### Characterization of Best1-mediated glutamate conductance from hippocampal CA1 astrocytes

**Figure 1:**
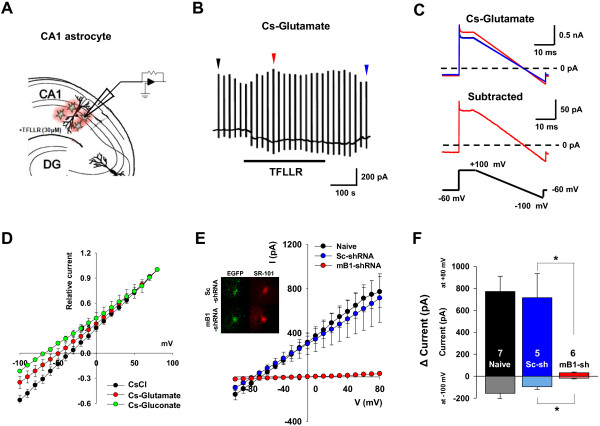
**Identification of TFLLR-induced anion conductance from hippocampal CA1 astrocytes. A**. Schematic diagram of whole-cell patch clamp recordings from astrocytes in hippocampal CA1 stratum radiatum region for measuring Ca^2+^-induced glutamate conductance from CA1 astrocytes. TFLLR-NH_2_ peptide (TFLLR) was used as PAR-1 agonist to induce increase in [Ca^2+^]_i_ in CA1 astrocytes. **B**. Representative current responses by voltage ramp command (from +100 mV to -100 mV) in hippocampal CA1 astrocyte. Arrows indicate ramp current before TFLLR treatment (black), after TFLLR treatment (red), and after washing (blue). **C**. Representative ramp current before TFLLR treatment (black trace), after TFLLR treatment (red trace), and after washing (blue trace). Dotted black line indicates 0 pA. **D**. Normalized average I-V relationships from whole-cell voltage clamp measurements of TFLLR-induced currents with various anions such as CsCl (n = 6), Cs-glutamate (n = 8), or gluconate (n = 7) in pipette solution. Experiment was performed in the presence of gap junction blocker 100 μM of carbenoxolone. **E**. Averaged I-V curves recorded from naïve, scrambled-shRNA- (sc-shRNA) or mBest1-shRNA (mB1-shRNA)-expressing astrocytes. Inset: the representative images showing lentiviral-shRNA and EGFP- expressing astrocytes stained with astrocyte marker dye (SR-101). SR-101 and EGFP-stained hippocampal CA1 astrocytes were patch clamped with 140 mM glutamate-containing pipette solution and TFLLR peptide was used to induce CAAC current. **F**. Bar graph representing averaged current amplitude at V_h_ = 80 mV or - 100 mV. Numbers on each bar indicate number of astrocytes from hippocampal slices of at least two mice. *p < 0.05.

To identify the anion channel-mediated glutamate conductance from astrocytes in hippocampal slices, we utilized PAR-1 as a tool for eliciting Ca^2+^-dependent anion conductance from hippocampal astrocytes. Previously, it has been demonstrated that both in cultured astrocytes and astrocytes in hippocampal slice, PAR-1 activation by treatment of its agonist, TFLLR-NH_2_ peptide (30 μM of TFLLR), can induce Ca^2+^-activated anion current (CAAC), and this anion conductance is mediated by Best1 channel [4,7]. We tested whether Best1-dependent anion conductance is carried by glutamate. A whole-cell patch clamp was made onto hippocampal CA1 astrocytes labeled with sulforhodamine-101 (SR-101) [7]. To reduce the voltage-clamp error due to electrical coupling among neighboring astrocytes through gap junction [10], we performed the experiment with the slices pretreated with a gap junction blocker, 100 μM carbenoxolone for at least 30 minutes. Cl^-^, glutamate, or gluconate was included as a predominant anion of the pipette internal solution as previously described [7,11] to compare the permeability of each anion. The changes in conductance were monitored by a series of periodic voltage ramps from +100 mV to -100 mV applied before and during TFLLR application (Figure 1A, B) and by subtracting ramp currents recorded during TFLLR application from those measured before TFLLR application to obtain the I-V relationship of the TFLLR-induced CAAC (Figure 1C). The normalized I-V relationships from our recordings showed that substituting glutamate and gluconate with the intracellular Cl^-^ resulted in a significant inward current at the holding potential of -100 mV (Figure 1D), indicating a significant efflux of glutamate and gluconate at this potential [12].

We next tested whether this astrocytic glutamate conductance is mediated by Best1. In our previous report, we demonstrated that over 90% of GFAP positive astrocytes in *stratum radiatum* of CA1 hippocampus express functional Best1, and this Best1 mediates the TFLLR-induced CAAC [7]. Thus, it is plausible to hypothesize that Best1 is also responsible for glutamate-mediated conductance elicited by PAR-1 activation. To reduce the Best1 expression in the astrocytes, we utilized the Best1-sensitive short hairpin RNA (shRNA) [7]. A whole-cell patch clamp was made onto the astrocytes expressing scrambled- or Best1-shRNA (identified by co-staining of SR-101 and GFP (co-expressed with shRNA)) using the pipette solution containing glutamate as a main anion (Figure 1E). We found that TFLLR-induced anion conductance in Best1-shRNA expressing astrocytes was almost completely eliminated, compared to scrambled-shRNA expressing astrocytes (Figure 1E, F). The effect on TFLLR-induced glutamate efflux was quantified by measuring current magnitude at -100 mV: glutamate efflux at this potential was significantly smaller in astrocytes from mice injected with Best1-shRNA than with scrambled-shRNA or from naïve mice (Figure 1F). Taken together, our data indicate that Best1 in hippocampal astrocytes is required for generating TFLLR-induce anion conductance carried by glutamate.

### Astrocytic glutamate conductance is unaffected by gap junction hemichannel or Ca^2+^-activated K^+^ channel

**Figure 2:**
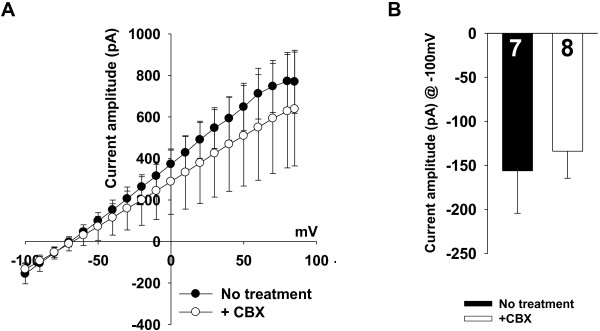
**TFLLR-induced conductance is independent on activation of gap-junction hemichannels and Ca**^**2+ **^**activated potassium channel. A**. Averaged current–voltage relationships from TFLLR-induced current using Cs-Glutamate pipette solution in the presence of gap junction blocker (+CBX) or normal ACSF bath solution (No treatment). **B**. Bar graphs indicates the averaged current amplitude at holding potential of -100 mV (Current amplitude @ -100 mV) to test whether glutamate-mediated current in astrocytes is reduced by inhibiting the gap junction activity. p = 0.7 for Current amplitude @ -100 mV; unpaired t-test.

We performed sets of control experiments to test whether Best1-dependent glutamate conductance is a genuine glutamate and to eliminate possible contributions of other channels to the TFLLR-induced conductance. Because of the abundant expression of gap-junctions between astrocytes and its possible contribution to our measurement, we compared TFLLR-induced glutamate conductance in the presence or absence of carbenexolone (CBX; 100 μM). It has been known that CBX blocks electrical signal through gap junction in astrocytes [13-16]. Firstly, we measured Rm value of hippocampal astrotytes in the presence or absence of CBX. Rm value was slightly increased from 9.56 ± 1.814 MΩ (mean ± s.e.m; N = 10; in the absence of CBX) to 14.77 ± 2.74 MΩ (N = 9; in the presence of CBX) by CBX treatment, but this change was not significant (p = 0.1250, unpaired t-test, data not shown). This insignificant change in the membrane resistance by CBX treatment is probably due to the astrocytes’ leaky property (usually Rm is about 10 mega ohm), resulting in the length constant-mediated voltage clamping error. Furthermore, our data showed that there is no gap-junction dependent change in glutamate conductance in our experimental condition (Figure 2A, B) indicating no role of gap-junction mediated mechanism in generating astrocytic glutamate conductance.

**Figure 3:**
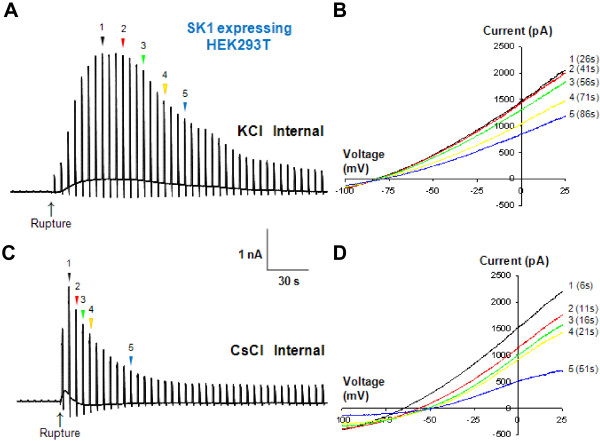
**TFLLR-induced conductance is mediated by Best1 channel in hippocampal CA1 astrocytes. A**. Representative current responses induced by whole-cell patch clamp from HEK293T expressing hSK1 using KCl-containing pipette solution (n = 8). The current responses were recorded in response to a voltage ramp command (from -100 to +100 mV, 1 s duration, 0.2 Hz; V_h_ of -60 mV. Internal pipette solution is composed of (mM) 150 KCl, 10 HEPES, 1 CaCl_2_, 1 MgCl_2_, and 5 EGTA (100 nM free Ca^2+^). **B**. The 5 selected representative current–voltage relationships recorded from a). **C**. Representative current responses induced by whole-cell patch clamp from HEK293T expressing hSK1 using KCl-containing pipette solution (n = 7). **D**. The 5 selected representative current–voltage relationships recorded from c). Averaged time that Er reaches to plateu from rupture is 63.4 ± 14.6 s (n = 7).

Next, it is still possible that Ca^2+^-activated K^+^ channel contributes to the TFLLR-induced conductance from astrocytes. The major cation in the pipette solution is Cs^+^ which is known to inhibit the most of current mediated by Ca^2+^-activated K^+^ channel, SK1 channel [17]. To directly test whether Cs^+^ based internal pipette solution blocks Ca^2+^ activated K^+^ channel, and to show that Ca^2+^-activated K^+^ conductance does not contribute to TFLLR-induced conductance, we expressed SK1 channel in HEK293T cells and measured the SK1-mediated whole-cell current induced by Ca^2+^-included pipette solution. When the major cation was K^+^ ion in the pipette solution, Ca^2+^-induced K^+^ conductance was rapidly evoked upon whole-cell rupture and slowly desensitized, but still remained even several minutes after the whole-cell rupture (Figure 3A, B). By contrast, when Cs^+^-containing pipette solution was used, Ca^2+^-induced K^+^ conductance by whole-cell rupture of SK1 expressing HEK293T cell was disappeared within ~60 sec (Figure 3C, D), eliminating the possible contribution of Ca^2+^-induced K^+^ conductance to our TFLLR-induced glutamate current. This is consistent with our previous study showing that the size of TFLLR-induced CAAC currents recorded using K^+^ based pipette solution is similar with that using Cs^+^[7].

### Dual patch-clamp recording from a single astrocyte reveals voltage error

**Figure 4:**
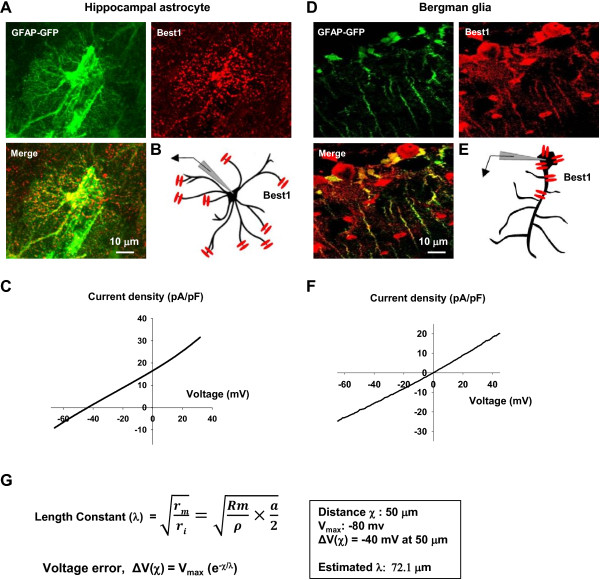
**Reversal potential of Best1-mediated anion conductance in hippocampal astrocyte and bergman glial cell. A**. Immunohistochemical staining of GFAP-GFP (green), Best1 (red), and merged image showing that Best1 channels are exclusively expressed in microdomain of hippocampal astrocyte. **B**. Schematic diagram of Best1 expression in hippocampal astrocytes. **C**. The representative I-V relationship of NPPB sensitive current showing anion conductance in hippocampal astorcytes. **D**. Immunohistochemical staining of GFAP-GFP (green), Best1 (red), and merged image showing that Best1 channels are exclusively expressed in soma of bergman glia. **E**. Schematic diagram of Best1 expression in bergman glia. **F**. The representative I-V relationship of NPPB sensitive current showing anion conductance in bergman glia. **G**. The formula of length constant in hippocampal astrocytes.

To determine the glutamate permeability ratio, we examined the reversal potential from the I-V relationships that we obtained in Figure 1B. Surprisingly, the observed reversal potential of the intracellular Cl^-^ condition (-34.8 mV) deviated significantly from the predicted 0 mV according to the Nernst equation under symmetrical Cl^-^. This was markedly different from the reversal potential obtained from Bergmann glial cells under similar recording and symmetrical Cl- condition [6]: the reversal potential of the Best1-mediated CAAC current from Bergmann glial cell was similar to the predicted value of 0 mV (Figure 4F) [6]. The reversal potential was clearly shifted by substitution of intracellular anions [shifted from -34.8 mV (Cl^-^) to -49.1 mV (glutamate) and -68.1 mV (gluconate)] (Figure 1B). The shift in reversal potential by ion substitutions indicates that glutamate is less permeable than Cl^-^ but more permeable than gluconate, as predicted by the size of the anions [12]. The deviation in the observed reversal potential from the symmetrical Cl^-^ condition raised a possibility that voltage errors can be substantial under conventional single-electrode patch-clamp configuration due to astrocyte’s inherent low membrane resistance [10].

**Figure 5:**
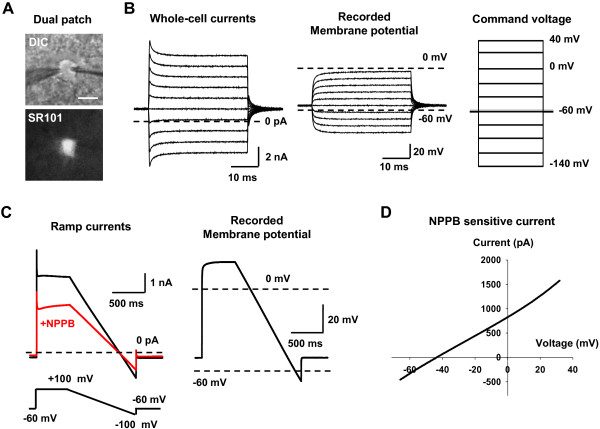
**Dual-patch clamp from hippocampal astrocyte reveals the voltage error that accounts for the shift in reversal potential of the Ca**^**2+**^**-activated anion conductance. A**. DIC Image of single hippocampal astrocyte loaded with SR101, with two electrodes sealed on the cell somata. Scale bar: 10 μm. **B**. The whole cell current recorded from the right electrode and real membrane potential recorded from the left electrode. Command voltages were stepped from -140 mV to +40 mV with an increase of 20 mV. The holding potential was -60 mV. **C**. The ramp currents recorded from the right electrode and actual membrane potential recorded from the left electrode (Average of 5 sweeps). Ramp protocol was given from +100 mV to -100 mV. **D**. The representative I-V relationship of subtracted current between before and during NPPB application represents NPPB sensitive current.

To estimate the degree of voltage error due to astrocyte’s leaky membrane in our experimental condition, we next performed a dual patch-clamp recording onto CA1 astrocytes to observe the actual membrane voltage during the voltage clamp experiment. Experimentally, two recording electrodes were sealed consecutively to a single astrocytic soma (Figure 5A). One electrode was used to perform whole-cell voltage clamp recording, and the other to monitor the actual voltage change in current clamp mode without adding holding currents. Firstly, we recorded passive conductance during a series of step-voltage protocol in astrocytes (Figure 5B). We observed a significant discrepancy between the command voltage and the actual voltage (50% error at command voltage of +40 mV, Figure 5B), consistent with the previous report [10]. This discrepancy is mostly likely due to a voltage drop across the access resistance of the patch pipette, which is shown to be prominent especially when the membrane resistance is lower than the access resistance as in astrocyte [10]. However, this voltage error due to a voltage drop across the access resistance should not and did not affect the reversal potential of the passive conductance, which falls near the equilibrium potential for K^+^ ion at around -80 mV (Figure 5B) [10].

We then performed dual patch-clamp recording to isolate the CAAC current induced directly by Ca^2+^ in the patch pipette solution and by using the blocker, 5-Nitro-2-(3-phenylpropylamino)benzoic acid (NPPB) to obtain the NPPB-sensitive CAAC current under symmetrical Cl^-^ condition from a single astrocyte (Figure 5). This allowed us to isolate Best1-mediated conductance during the dual patch clamp recording and to confirm whether NPPB-sensitive current in this condition is same with the one recorded in Figure 1A. We isolated NPPB-sensitive current using voltage ramp protocol from +100 mV to -100 mV (Figure 5C) and plotted as an I-V relationship (Figure 5D). Instead of using the command voltage for the plot, we used the recorded actual voltage for the I-V relationship to bypass the voltage error due to access resistance (Figure 5D). The I-V relationship of Best1-medaited, NPPB-sensitive current showed a reversal potential of around -40 mV (Figure 5D), which was similar to the deviation that we observed with the reversal potential of TFLLR-induced CAAC current in Figure 1B (-34.8 mV).

### Deviation of reversal potential due to specific localization of Best1 in microdomain

What is the source of -40 mV deviation in the reversal potential of both NPPB sensitive current and TFLLR-induced CAAC current? The distortion of voltage-gated currents due to incomplete space-clamp in neuronal dendrites under patch-clamp technique has been extensively reported [18-25]. Unlike neurons, astrocytes are known to exhibit high expression of background potassium channels, which results in very low membrane resistance in a unit length, R_m_. In addition, astrocytes contain elaborate processes with a small diameter of around 1 μm, which should result in high axial resistance per unit length, R_i_. The combination of these two factors affects the length constant by the relationship, λ = $\sqrt{\frac{\mathrm{Rm}}{\mathrm{Ri}}}$, which governs the degree of voltage error (ΔV) due to an incomplete space clamp in voltage clamping experiments by the relationship, ΔV(χ) = V_max_ (e^-χ/λ^), where χ represents the distance from the recording electrode. The length constant λ can be rewritten as, λ = $\sqrt{\frac{\mathrm{Rm}}{p}}\times \frac{a}{2}$, where ρ is the specific resistivity of astrocytic process and *a* is the diameter of astrocytic process. In case of hippocampal astrocytes, λ should be very low due to low R_m_ and high R_i_ and therefore the voltage error, ΔV should be significantly high. Similar to an axon of mammalian neuron, the diameter of each astrocytic process is estimated to be around 1 μm [26]. Best1 is reported to be expressed mostly in the distal microdomains of astrocytes, near synaptic junctions, as shown in our previous study [4] as well as in Figure 4A. Thus, the average distance of Best1 from the recording electrode, χ, should be around 50 μm (Figure 4B). Under symmetrical Cl^-^ condition, the expected voltage at the microdomain when the Best1 is fully open should be around 0 mV. Thus, the V_max_ can be estimated to be around -80 mV (V_max_ = -80 mV – 0 mV), based on the fact that the resting membrane potential at soma around the recording electrode is around -80 mV. Using the observed voltage error of -40 mV for ΔV(χ) at 50 μm, we can now estimate the length constant λ for astrocytic processes to be 72.1 μm (or 0.0721 mm) (Figure 4G), which is much lower (44 fold less) than the reported length constant for neuronal dendrite (λ = 3.16 mm, [27]). Unlike hippocampal astrocytes, Bergmann glial cells express Best1 in the cell body as well as main processes (Figure 4D) [6]. Therefore, the voltage error is minimal due to minimal distance between the locations of Best1 to the recording electrode. This is most likely the reason for the consistency between the observed reversal potential and the predicted value in Bergmann glial cells (Figure 4D). Taken together, we concluded that the I-V relationships in Figure 1B are deviated by about -40 mV due to voltage error arising from incomplete space-clamp, which originates from the very low length constant of the astrocytic processes. These results and estimations strongly support the idea that functional Best1 channel is mostly expressed in distal microdomains of hippocampal astrocytes, as predicted by the immunohistochemical and electrophysiological evidence.

### Calculation of glutamate permeability ratio

The relative shift in the reversal potential might underestimate the true permeability ratio due to the evident voltage error. Nevertheless, we found a shift in reversal potential for each anion in accord with anion substitution in the pipette solution (Figure 1B). Based on the shift in reversal potential, it was possible to calculate a relative permeability of each anion to Cl^-^ anion according to the Goldman-Hodgkin-Kats equation (see Materials and Methods). The estimated permeability ratio of glutamate to Cl^-^ was P_glutamate_/P_Cl_ = 0.53, which was in the range of the reported values between 0.47 for cultured astrocytes [7] and 0.67 for heterologously expressed Best1 channels in HEK293T cells [4]. The calculated glutamate permeability ratio of Best1 was much higher than the reported GABA permeability ratio, P_GABA_/P_Cl_ = 0.19, of the Best1 in Bergmann glial cells of the cerebellum [6].

## Conclusion

In conclusion, our results indicate that Best1 in CA1 hippocampal astrocytes shows a relatively high permeability to glutamate. In addition, its specific localization at microdomain near synaptic junctions is further evidenced by the deviation of the reversal potential of Best1-mediated CAAC current. This unique biophysical property of Best1 makes this channel a prominent molecule responsible for channel-mediated release of important gliotransmitters such as glutamate and GABA. It is possible that Best1 channel is also permeable to many other important gliotransmitters such as D-serine, aspartate and taurine. Future studies will provide these intriguing possibilities of their roles in channel-mediated gliotransmitter release and in synaptic functions.

## Methods

### Cell culture and electrophysiology

HEK293T cell culture was performed as previously described [3]. For measuring SK1 conductance, HEK293 cells were transfected with human SK1-containing plasmid pcDNA3.1-hSK1 using Effectene (Qiagen, Valencia, CA, USA) at 1 mg of DNA per 35-mm culture dish. Transfected HEK293 cells were recorded using a conventional whole-cell patch-clamp within 48 hrs. Fire-polished borosilicate glass patch pipettes were 3 ~ 5 MΩ. Experiments were conducted at room temperature (20 ~ 25°C). Current–voltage curves were established by applying 500 ms duration voltage ramps from -100 to +100 mV (V_h_ = -70 mV). Data were acquired with an Axopatch 200A amplifier controlled by Clampex 10.0 software via a Digidata 1322A data acquisition system (Molecular Devices, Sunnyvale, CA, USA). The extracellular solution is composed of (mM) 150 NaCl, 10 HEPES, 3 KCl, 2 CaCl2, 2 MgCl2, and 5.5 glucose at pH 7.3 adjusted with NaOH (300 ~320 mOsm). Internal pipette solution is composed of (mM) 106 CsCl or KCl, 20 TEA-Cl, 10 BAPTA, 8.7 CaCl_2_, 0.5 MgCl_2_, and 5 HEPES (500 nM free Ca^2+^).

### Production of mBest1-shRNA containing lentivirus and delivery into mouse hippocampus

Scrambled shRNA and *mBest1*-shRNA were inserted into pSicoR lentiviral vector (provided by Dr. T. Jacks through Addgene Inc.) [28] as previously described [27]. For shRNA expression into hippocampal CA1 region, lentivirus (produced by Macrogen, Korea) was delivered into hippocampal CA1 region of wild type C57BL/6 mice by a stereotaxic surgery method [29].

### Slice preparation and electrophysiology

Horizontal or transverse mouse brain slices (300 ~ 400 μm) containing hippocampus were acutely prepared as previously described [3]. SR-101 (Sigma; 1 μM) dye was loaded into acute brain slice as previously described [30]. Prepared slices were left to recover for at least 1 h before recording in oxygenated (95% O_2_ and 5% CO_2_) artificial cerebrospinal fluid (ACSF; in mM, 130 NaCl, 24 NaHCO_3_, 3.5 KCl, 1.25 NaH_2_PO_4_, 1 CaCl_2_, 3 MgCl_2_ and 10 glucose, pH 7.4.; room temperature). The standard ACSF recording solution was composed of (mM): 130 NaCl, 24 NaHCO_3_, 3.5 KCl, 1.25 NaH_2_PO_4_, 1.5 CaCl_2_, 1.5 MgCl_2_ and 10 glucose saturated with 95% O_2_–5% CO_2_, at pH 7.4. To observe Ca^2+^-induced glutamate conductance in CA1 astrocytes, the pipette solution contained (in mM) 133 Cs-glutamate, 1 MgCl_2_, and 10 HEPES, at pH 7.3 adjusted with CsOH (~280 Osm). For Cl^-^ or gluconate-based internal solution, Cs-glutamate was replaced with the same concentration of CsCl or Cs-gluconate. To block the effect of neuronal spontaneous activity on astrocytes, TTX (0.5 μM; Tocris) was added into ACSF. Experiments with a holding current more than -100 pA or a change in input resistance >30% of control were rejected. To induce CAAC activation from CA1 astrocytes, 30 μM of TFLLR was bath-treated. In one series of experiments, dual-patch recording from a single astrocyte was used to directly measure the voltage errors in whole-cell voltage clamp recording. The standard electrode solution contained the following (in mM): 140 KCl, 0.5 CaCl_2_, 1.0 MgCl_2_, 5 EGTA, 10 HEPES, 3 Mg-ATP, and 0.3 Na-GTP (pH 7.3). The pH was adjusted to 7.3 with KOH.

Length constant (λ) = $\sqrt{\frac{\mathrm{Rm}}{\mathrm{Ri}}}$ (Rm is membrane resistance, and Ri is axial resistance.).

V(x) = Vmax(e^-x/λ^) (x is the distance from the start of the potential).

### Immunohistochemistry

Rabbit polyclonal Best1 IgG was produced using antigen (Ab Frontier). Primary antibody was then applied at appropriate dilution [Chicken polyclonal GFP at 1:1000 (Abcam), Best1 IgG at 1:100] and incubated overnight at 4°C. After this, the sections were washed three times in 0.1 M PBS and incubated in secondary antibody [DyLight 488 donkey anti-chicken IgG at 1:200 (The Jackson Laboratory), Alexa 555 donkey anti-goat IgG at 1:200 (Invitrogen)] for 2 h. After three rinses in 0.1 M PBS and DAPI staining at 1:1000 (PIERCE), the sections were mounted on polysine microscopic slide glass (Thermo Scientific). Images were acquired using a Nikon A1R confocal microscope.

### Statistical analysis

Statistical analysis was performed using Prism software (GraphPad software). Statistical significance between two groups was analyzed by using student t-test and among multiple groups at least three groups by using One-way ANOVA with Dunnett’s multiple comparison. All values are shown as mean ± s.e.m.

## Competing interests

The authors declare that they have no competing interests.

## Authors’ contributions

HP performed and designed the experiments, performed a whole-cell voltage clamp of hippocampal CA1 astrocytes, and wrote the manuscript. KSH performed a dual patch clamp onto hippocampal astrocytes, and wrote the manuscript. SJO performed a whole cell patch clamp recording for SK1 channel. JS performed a whole-cell voltage clamp of hippocampal CA1 astrocytes. BEY and SJ performed immunohistochemistry. CJL designed the experiments and wrote the manuscript, and supervised the entire project. All authors read and approved the final manuscript.
